# The photon beamline vacuum system of the European XFEL

**DOI:** 10.1107/S1600577521005154

**Published:** 2021-06-08

**Authors:** Martin Dommach, Massimiliano Di Felice, Bianca Dickert, Denis Finze, Janni Eidam, Nicole Kohlstrunk, Maik Neumann, Frederik Meyn, Michaela Petrich, Benoit Rio, Harald Sinn, Raúl Villanueva

**Affiliations:** a European XFEL, Holzkoppel 4, 22869 Schenefeld, Germany

**Keywords:** photon beamlines, vacuum, beam transport system, XFEL

## Abstract

The photon beamline vacuum system of the European X-ray Free-Electron Laser Facility (European XFEL) is described. The ultra-large, in total more than 3 km-long, fan-like vacuum system, consisting of three photon beamlines is an essential part of the photon beam transport. It is located between the accelerator vacuum system and the scientific instruments. The main focus of the design was on the efficiency, reliability and robustness of the entire system to ensure the retention of beam properties and the operation of the X-ray optics and X-ray photon diagnostics components. Installation started in late 2014, the first of the three beamline vacuum systems was commissioned in spring 2017, and the last one was operational in mid-2018. The present state and experience from the first years of operation are outlined.

## Abstract 2

A description of the photon beamline vacuum system of the European XFEL is given, together with experiences gained during the first years of operation.

## 1. Introduction   

The European XFEL is a hard X-ray free-electron laser (X-ray FEL) with MHz repetition rate that started operation in April 2017 (Altarelli *et al.*, 2006 ▸; Decking *et al.*, 2020 ▸). The X-ray beam is delivered to the scientific instruments, which are located in the experiment hall at the end of the underground tunnels (Tschentscher *et al.*, 2017 ▸). Up to three instruments are located on each of the three photon beamlines.

The photon vacuum beamlines are designed for ultrahigh-vacuum (UHV) conditions with an average pressure lower than 1 × 10^−7^ mbar and a helium leak tightness of 1 × 10^−10^ mbar L s^−1^. A horizontal separation of 1.4 m is required for the vacuum pipes close to the end of the tunnel to have enough space for the scientific instruments in the adjacent experiment hall. At the hard X-ray beamlines, this leads to long vacuum pipe sections to bridge the distance of about 550 m between distribution mirror and instruments, as the deflecting angles are very small. The above-mentioned X-ray mirrors as well as other state-of-the-art X-ray optics (Sinn *et al.*, 2019 ▸) are very sensitive to hydrocarbon contamination and dust particles on the optical surfaces. As a bake-out of the entire vacuum system was considered too laborious, all components built into the vacuum beamline were specially cleaned before installation, to remove hydrocarbon-containing residuals from the manufacturing process. Components used in close proximity to (30 m before and after) mirrors and gratings are prepared and installed in cleanrooms to avoid particle contamination on the inner surfaces.

Access to the tunnels is usually limited to two long maintenance periods per year for more extensive interventions; therefore, we have placed special emphasis on the high availability, reliability and serviceability of the system. Wherever possible, moving parts and active cooling of vacuum devices have been avoided.

**Figure 1:**
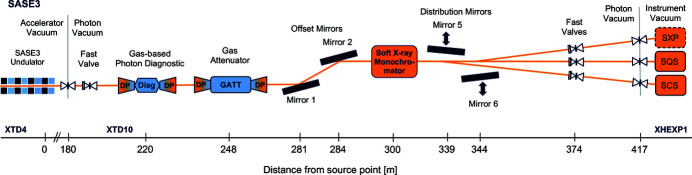
Schematic overview of the SASE3 photon vacuum system. UHV sections are indicated in orange colour.

To reduce the photon beam intensity of the soft X-ray beamline SASE3 (Fig. 1 ▸ [Sec sec1]) in a controllable way, a gas attenuator device has been developed and installed. It technically allows up to 35 mbar nitrogen or argon in its active gas cell, which is connected windowlessly to the beamline vacuum system using a high-performance differential pumping design. More information on the gas attenuator can be found in Section 4.

## 2. Vacuum system design   

### 2.1. Materials   

Stainless steel AISI 316L and AISI 304L are mainly used as vacuum pipe and vessel material for the whole beam transport system. The roughness of the inner surface of all vacuum components was specified as 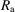 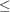 0.8 µm. All UHV flanges are of the knife-edge type, according to the ISO 3669 standard, with some exceptions for chambers with large diameters, like mirror chambers, where DN600 COF flanges have been used, or some rectangular vessels, like the SASE3 soft X-ray monochromator chamber, which are sealed by utilizing an aluminium wire. Knife-edge flanges were made from AISI 316LN ESR stainless steel and sealed by using an oxygen-free high-conductivity (OFHC) copper gasket. The entire system is built in a way that only all-metal seals are used to separate vacuum from atmospheric environment.

### 2.2. Parts cleaning and ‘particle-free’ areas in the beamline   

All large vacuum parts made from stainless steel, such as chambers or long pipe segments, were cleaned before installation to minimize the amount of hydrocarbons in the residual gas following this procedure:

(1) Chemical mechanical precleaning (based on HNO_3_).

(2) Rinsing with demineralized water.

(3) Anodical cleaning (based on H_2_SO_4_ and H_3_PO_4_).

(4) Rinsing with demineralized water.

(5) Chemical passivation of the surface (based on HNO_3_).

(6) Rinsing with demineralized water.

(7) Final treatment in cleanroom (based on ISO 14644, level ISO 5).

 (*a*) Rinsing with 80°C demineralized water.

 (*b*) Drying with nitrogen.

 (*c*) Quality control (visual inspection, surface roughness).

 (*d*) Closing with clean plastic flange caps.

The cleaning of large parts was done by an external company and supplied ready for installation. Small parts were cleaned in-house, using a high-throughput lab washing machine (Belimed WD 290) that can be unloaded directly to a cleanroom. This machine is operating with demineralized water and uses alkaline cleaning detergents (Dommach, 2015 ▸).

Certain areas of the beamline require a reduced number of particles inside the vacuum chamber to avoid destroying the unique parameters of the X-ray FEL beam, like wavefront and coherence properties, by contamination of the optical surface of X-ray mirrors or gratings. Therefore, so-called ‘particle free’ areas were defined 30 m around these optical elements and 30 m towards the interface to the scientific instruments, which also operate sensitive beam focusing mirror systems. Here, we aim for an air cleanliness by particle concentration level ISO 5 according to the ISO 14644 standard. Wherever possible, vacuum parts installed in this part of the beamline were cleaned using a wet cleaning process followed by a cleanroom-compatible treatment. If this procedure was not possible (*e.g.* vacuum gauges, fast-closing valves, gate valves and complex mechanics, which could not be dismounted for cleaning), the particles were removed using an ionized nitrogen blowing gun in a cleanroom under permanent monitoring by a particle counter. This procedure was carried out until the displayed particle concentration corresponded to the ISO 5 level. Special care has been taken to put the NEG cartridge pumps into operation, following a procedure of multiple cycles of conditioning and activation to lower the particle release to an acceptable level before installation, described by DESY (Lederer *et al.*, 2017 ▸).

**Figure 2:**
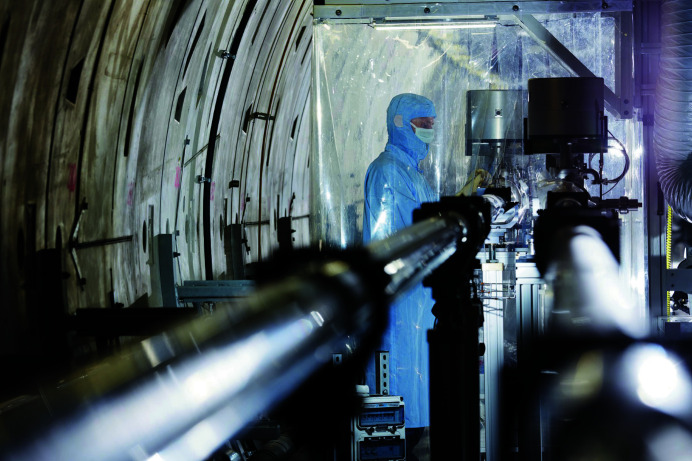
Beamline assembly under cleanroom conditions (Credit: European XFEL / Heiner Müller-Elsner).

Assembling the particle-free vacuum sections in a harsh environment like the European XFEL underground tunnels was a challenging task, as the whole installation was performed taking advantage of small mobile cleanrooms consisting of a filter-fan unit and a cleanroom curtain (Fig. 2 ▸ [Sec sec2.2]). Wherever possible, components were pre-assembled in a well monitored cleanroom laboratory to minimize the number of flange connections to be made in the tunnel.

### 2.3. Design of long vacuum sections   

To reduce the number of flange connections, potential leaks and therefore installation work, 18 m-long DN100 beampipe sections were built of AISI 316L stainless steel pipes, which were welded together in the tunnel from three 6 m-long prefabricated pipe segments. After manufacturing, the segments were brought to a cleaning company before delivery ‘ready for installation’ to the European XFEL. The manufacturing company performed the final assembly in the photon tunnels XTD6 and XTD9 by orbital Tungsten Inert Gas (TIG) welding. About 100 pieces of the 18 m-long vacuum pipes were produced.

### 2.4. Vacuum components   

To obtain the required pressure, different types of vacuum pumps are used. For permanent pumping, sputter ion pumps (SIPs) of StarCell (Agilent Technologies) type are used, as they are easy to operate and almost maintenance-free. The archived base pressure at the pumps, usually in the 10^−9^ mbar range, is considerably better than the average pressure to guarantee the expected lifetime of the pumps, which is indicated by the manufacturer as 80000 h at a pressure below 1 × 10^−6^ mbar.

In total, about 300 sputter ion pumps, 50 turbomolecular pumps, 15 NEG cartridge pumps and 23 permanently operated scroll and roots type roughing pumps are installed at the three beamlines. All installed turbomolecular pumps can be separated from the beamline vacuum by a gate valve to enable the possibility of a quick exchange of the pump for regular maintenance without venting the whole vacuum section and to automatically detach it by the vacuum interlock system in case of malfunction of the pump.

The NEG cartridge pumps, made of ZrVFe-alloy with 1000 L s^−1^ pumping speed, are installed on locations that are sensitive to hydrocarbon contamination, like mirror chambers.

The gas composition at mirror chambers is monitored using residual gas analyser (RGA) devices; these are installed using a 3 m-long extension cable to separate analyser head and electronics unit to avoid radiation damage.

All manually operated valves for roughing and venting are of the all-metal sealed type. The gate valves separating individual vacuum sections or permanently operated turbomolecular pumps from the beamline vacuum have FKM elastomer seals, as they remain open during normal beam operation; exceptions to the sealing type are all-metal sealed gate valves at the interfaces to the accelerator and to the scientific instruments.

### 2.5. Segmentation   

To minimize the impact of a vacuum failure, to allow easy maintenance and to shorten the pump downtime, individual vacuum sections can be separated by gate valves. In total, 90 vacuum sections were implemented at the three beamlines. Some of these sections are relatively short (*e.g.* a few metres around an optical component that might require frequent interventions or maintenance). Other sections (*e.g.* the beam transport pipe between distribution mirror and scientific instruments at the hard X-ray beamlines SASE1 and SASE2) are up to 200 m long. Each vacuum section is equipped with:

(i) One venting unit, including a manual DN16 all-metal valve and a 0.5 µm particle filter.

(ii) At least one manual DN40 all-metal roughing valve for pump cart connection.

(iii) A set of vacuum gauges (Pirani and cold cathode type) to be able to measure up to atmospheric pressure during pump-down and venting and to have a redundant pressure reading in conjunction with the sputter ion pumps for interlock purposes.

(iv) At least two sputter ion pumps.

This segmentation allows us to meet the envisaged short pump-down time. The recovery of a vacuum section, including pump-down with one or more mobile pump carts, leak test and commissioning of ion pumps, usually takes a maximum of 24 h.

### 2.6. Fast-closing valves   

To secure the operation of the beam transport vacuum system, needed to guide the X-ray beam to one scientific instrument, in the extremely rare case of a sudden air inrush, DN100 fast-closing valves of the flapper type are installed 27 m behind the separation chamber; downstream from that vacuum vessel, the X-ray beam is guided into two separate vacuum pipes leading to the scientific instruments. If the fast-closing valve of one instrument branch is triggered, the other instrument of this beamline remains operational. These valves are triggered by a redundant system of two cold cathode sensors, which are located 30 m away from the fast-closing valve, in order to have the valve controller react fast enough.

At the windowless interfaces to the accelerator vacuum system (Dommach *et al.*, 2018 ▸), the diameter of the beampipe is considerably smaller; DN40 fast-closing valves of the linear actuator type could be installed, which can be triggered by a pair of redundant sensors on each side. These valves have the advantage of working in both directions, so the accelerator vacuum is protected from a rapid pressure increase coming from the photon vacuum side, and vice versa.

All fast-closing valve controllers are directly connected to the machine protection system (MPS) that revokes the beam permission within 25 µs to prevent the valves from being hit by the photon beam. The closing time of the fast valves is on the order of 10 ms; the trigger level is set to 5 × 10^−6^ mbar.

## 3. Differential pumping for gas-based beam diagnostics   

**Figure 3:**
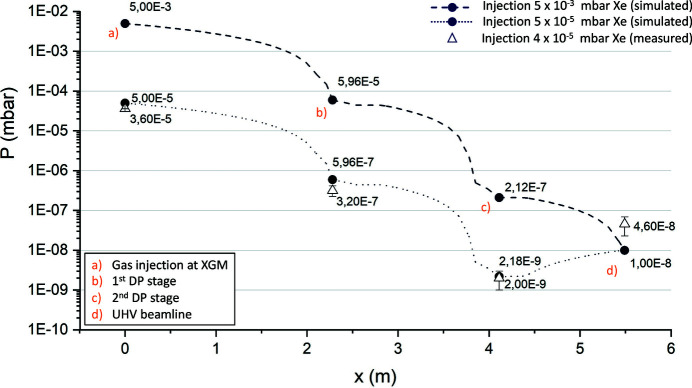
Simulated pressure profiles, compared with measured data of the two-stage DP module.

The use of non-invasive, gas-based beam diagnostics devices is central to the evaluation, adjustment, and control of the photon beam properties. In some places, where gas-based photon diagnostic components are installed, it is not possible to operate with sputter ion pumps. All three beamlines are equipped with X-ray gas monitors (XGMs) (Grünert *et al.*, 2019 ▸; Maltezopoulos *et al.*, 2019 ▸), which require a permanent supply of gas. Depending on each specific operation requirement (*e.g.* photon beam wavelength, intensity), these devices are operated with a continuous injection of noble gases, typically xenon or krypton, but operation with argon, neon or nitrogen is possible. Upstream and downstream of the X-ray gas monitors, continuous differential pumping (DP) stages with 25 mm apertures, consisting of turbomolecular pumps and oil-free scroll pumps, are installed to ensure proper pumping of the introduced gas and to obtain UHV conditions in adjacent sections. The well established operation range at the injection volume encompasses from 1 × 10^−7^ mbar up to 1 × 10^−4^ mbar. Provided that most of the species are noble gases, the use of a kinetic vacuum pumping mechanism is the most reasonable choice in terms of performance, compared with the use of sputter ion pumps anywhere else in the European XFEL photon beam transport vacuum system. Due to the need of a windowless beam transport system, a differential pumping scheme is mandatory to provide the required decoupling between the mentioned operative pressure scenarios and the UHV conditions in the adjacent vacuum sectors. A performance requirement of at least four orders of magnitude in the pressure level reduction is considered to minimize the residual process gas flow to the rest of the beam transport system. Simulated pressure profiles, taking into account the worst case (injection of 5 × 10^−3^ mbar of xenon) and the usual working point for the XGM (injection of 5 × 10^−5^ mbar of xenon), compared with measured data for a working point of 4 × 10^−5^ mbar xenon are shown in Fig. 3 ▸. The pressure set point at the XGM location (*a*) is precisely measured with a spinning rotor gauge (SRG), and the gas is expanded in the diagnostics chamber using a motorized dosing valve. The data measured at the DP stages (*b*) and (*c*) are total pressure readings from cold cathode gauges, without any correction factor applied. The total pressure value measured at the interface to the UHV beamline (*d*) was taken using a hot cathode gauge, also without application of a correction factor.

Given the total number of gas-based diagnostic devices along the European XFEL photon beamlines and the diverse installation features in some of them (*i.e.* beam axis is 2.6 m above the XTD2 tunnel floor level), a modular, flexible and adaptive solution, mostly based on ‘off-the-shelf’ UHV components, has been developed (Villanueva Guerrero *et al.*, 2016 ▸).

Following this purpose, the deployed differential pumping systems are based on a reduced number of building blocks:

(i) A unified pumping chamber design that can be mounted in any direction around the beamline longitudinal axis.

(ii) Standard turbomolecular pumps with a common fore vacuum system.

(iii) The use of narrow, long pipes (*L*/*D* < 30) as flow-limiting elements between pumping stages since the available longitudinal dimension is not particularly restrictive in the long photon tunnels.

For the particular case of the necessary conductance limitation, generalizing a large optical clear aperture of 25 mm between pumping stages became one of the most challenging design parameters. The safe use of these flow restriction elements is enabled by the design and installation of the adequate collimating boron carbide (B_4_C) aperture element located upstream from the differential pumping system, preventing the elements from being hit by the X-ray beam.

A total of ten differential pumping modules have been successfully installed around each of the defined gas sections (one at each side, namely ‘upstream’ and ‘downstream’). The performance results obtained during the test phase were also confirmed in the field installations. This particular validation was performed with the use of RGA measurements at an immediately adjacent sector, showing that the expected concept performance provides an effective decoupling between the average UHV beampipe pressure level and the required gas process conditions in the diagnostics modules.

## 4. SASE3 gas attenuator   

**Figure 4:**
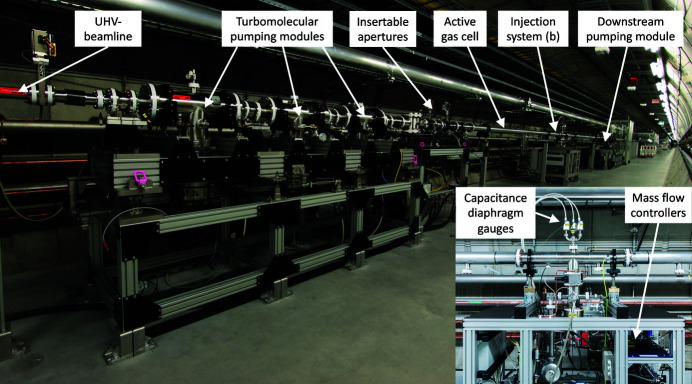
Photograph of the gas attenuator with the upstream differential pumping module visible in the front. The total length of the device amounts to 25 m. Inset: photograph of central gas injection module.

The SASE3 gas attenuator (GATT) is a non-conventional vacuum system when compared with the rest of the European XFEL beamline vacuum architecture, see Fig. 4 ▸. Conceptually, it is intended as a pressure-controlled leak of high purity gas, with the final goal of precisely controlling the transmitted intensity of the photon beam, yet without compromising the ultrahigh-vacuum base pressure of the adjacent sectors.

This particular gas-based device is the most appropriate alternative to conventional solid foils attenuators for the soft X-ray region. Attenuation foils would have to be very thin (*t* < 20 µm) and would not withstand the beam power due to heat transfer issues. A major difference, when compared with the vacuum concept of the gas-based photon diagnostics sectors, is the dramatic increase of the reference working pressure, as the gas attenuator is usually operated in the millibar range. This represents an average increase of five (or more) orders of magnitude in the gas flow to control at the injection location. The gas attenuator differential system shares the same principle of the differential pumping of the gas-based photon diagnostic sectors: the use of turbomolecular pumping modules, specifically designed and sized to decouple up to nine orders of magnitude pressure difference between the so-called ‘active gas cell’ and the interfacing UHV beamline elements.

In order to enhance precision, repeatability and speed, the injection system is based on an array of mass flow controllers. They are chosen in such a way that the necessarily very large dynamic range for the injected flows is also achieved. For the pressure measurement, a similar approach is developed by setting up an array of gas species-independent sensors (capacitance diaphragm gauges), enabling the use of the most precise gauging system for the range of interest (10^−4^ mbar to 100 mbar).

The device uses a variable aperture concept based on three different nominal diameters: 20 mm for the so-called ‘static’ apertures and 12 mm and 6 mm, respectively, for the discrete apertures that can be inserted on demand. The static types are basically implemented using short in-vacuum tubes which are permanently protected by a 16 mm B_4_C collimator located upstream in the beamline. The other two sets of apertures are instead produced directly as B_4_C orifice-plate disks. The smaller diameter apertures are necessary to limit the gas flow towards the differential pumping modules for those operational scenarios where higher attenuation level (hence higher pressure in the active cell) is required. The sequential insertion of these smaller apertures is possible because both beam divergence and cross section diminish as the photon energy increases. Therefore with a careful estimation of the minimum beam clear aperture versus photon energy, the gas attenuator can progressively operate in higher pressure levels without compromising either the beam passage or the performance of the differential pumping scheme.

Because this system is devoted to fulfil the daily requirements of both SASE3 instruments, a big effort has been made to facilitate its usability by means of an appropriate software interface. The combination of a refined safety programmable logic controller (PLC) interlock concept, the progressive implementation of a specific state machine-based algorithm for the automation of tasks, and the design of a user-friendly Karabo control panel have confirmed in the last three years of operation as a very successful solution. Some of the most relevant device characteristics are summarized in Table 1 ▸.

For a more detailed description, see Villanueva Guerrero (2021 ▸); for details of the initial specifications, see Sinn *et al.* (2012 ▸) and Villanueva Guerrero (2014 ▸).

## 5. PLC controls and the vacuum interlock system   

The control system hardware is a commercial PLC system from the company Beckhoff Automation. The central processing units (CPUs), an industrial PC, for each system are installed in electrical cabinets in the experiment hall; they are thus spatially separated from the tunnel.

The signal carrying wires from the beamline hardware are guided into ∼20 electrical cabinets in each beamline. The cabinet for the CPU and the cabinets in the tunnels are all connected to each other via EtherCAT bus-coupler and up to 100 m-long Ethernet cable or, for longer distances, via fibre optics, in a closed-loop structure. One loop consists of one CPU in the experiment hall and one or more terminal crates in the tunnel cabinets. In each tunnel, there is one loop for the vacuum system that is separated from other control system loops. The loops themselves can each contain more than 200 terminals of different types; numbers of the main hardware are given in Table 2 ▸.

Inside the tunnel cabinets, the electronic equipment is protected from damage caused by radiation during the FEL operation. For each PLC loop, a crate with the incoming and outgoing EtherCAT connection is mounted and contains all needed terminals for the different signal types (*e.g.* AI, DI, DO, serial port). Even in case of failure in one of the crates, all other crates can still be operated normally, due to the loop structure.

The typical devices in the vacuum control system part are:

(i) Pumps (sputter ion pumps, turbomolecular pumps, scroll pumps, multi-stage roots pumps, membrane pumps).

(ii) Valves (gate valves, angle valves, fast-closing valves, gas-dosing valves, mass-flow controllers).

(iii) Vacuum gauges of different types.

(iv) Sensors for monitoring compressed air pressure, cooling water flow, temperatures, *etc*.

(v) Multiple digital and analogue inputs and outputs.

To protect the whole system, an interlock logic is applied. For all interlock purposes, the most reliable and robust signals are used:

(i) For standard UHV sections, relay output from sputter ion pump controllers are closed only if the detected pressure is below 5 × 10^−6^ mbar (= setpoint).

(ii) For differential pumping sections, the relevant signals are analogue outputs from cold-cathode gauges.

The purpose of vacuum interlock is the following:

(i) If a critical situation is present, the section-separating gate valves in the section’s vicinity should be closed.

(ii) If a gate valve is closed and can be hit by the X-ray beam, the beam permission is deactivated via the MPS to avoid severe damage.

**Figure 5:**
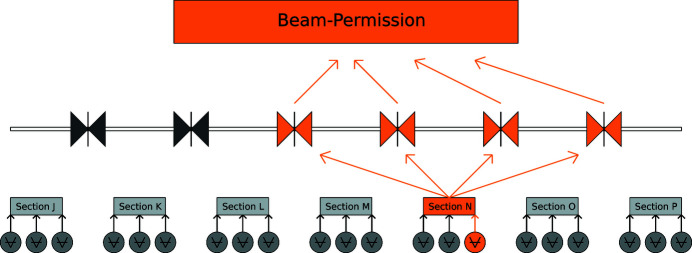
Interlock structure for the vacuum system: each section is ‘OK’ if all devices in it for pressure-detection are below 5 × 10^−6^ mbar; if not, it triggers the two gate valves at its borders to close in order to isolate the section, and the next gate valves in both directions (upstream and downstream) are closed as well. The beam permission is triggered to be ‘OFF’ if any of the gate valves in the beamline is closed.

The interlock logic (Fig. 5 ▸) is based on several stages:

(1) Individual elements in section are OK (pump/gauge below setpoint).

(2) Vacuum section is OK (all elements in section are in OK state).

(3) Gate valves are open.

(4) One of the following is true:

 (*a*) Beamline branch has all gate valves open and access for beam to the beamline branch is possible.

 (*b*) Beam access to the beamline branch is not possible (because the upstream photon beam-shutter is closed).

(5) Beam permission (from the vacuum system side) is granted.

Interlocks are defined on the PLC level, as this kind of system is considered more robust and stable than the below-mentioned software part of the control system.

The operation and control of the so-called gas sectors is particularly adapted to the specific operation of turbo­molecular pumps and roughing pumps (scroll type), and it is based on a ‘bottom-up’ logic approach, where their direct operation is permitted only when safely isolated from the beamline volume, with an automatic interlock if any operation error or abnormal operation condition is fetched by the PLC system. In such a way, this scheme allows a seamless integration on the general vacuum sector PLC interlock logic. Detailed documentation of the specific interlock concept for the gas attenuator has been given by Villanueva Guerrero (2020 ▸).

## 6. Control software   

The in-house-developed software *Karabo* (Hauf *et al.*, 2019 ▸) acts as interface between PLC and the user. From the control point of view, there are more complex devices like pumps and simpler devices like valves. For the first category, normally a serial interface is used for communication; for the second, it is typically a collection of a few signals that are merged into one device [*e.g.* a valve consists of two digital inputs (DIs) for state readout and one digital output (DO) for control to open or close the valve].

**Figure 6:**
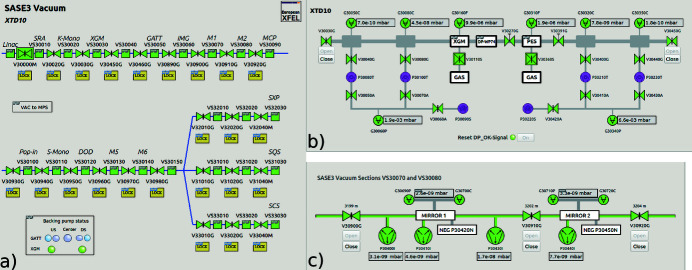
Screenshots taken from the *Karabo* GUI: (*a*) overview of the vacuum system in SASE3; (*b*) differential pumping stages for XGM in the tunnel XTD10; (*c*) detailed view of two sections (Mirror 1 and Mirror 2).

In the graphical user interface (GUI) of *Karabo* (Fig. 6 ▸ [Sec sec6]), graphical displays can be created that allow a clear overview of the system; buttons for the operation of devices facilitate the handling of the system for users.

To control a system of many devices, there is the option to create middle-layer devices. They are the instruments to allow controlled procedures of big systems in a safe and reliable way. This is in use especially for the SASE3 gas attenuator (Villanueva Guerrero, 2021 ▸).

Compared with the requirements of the whole European XFEL, the requirements of data storage for vacuum purposes are small. However, the data are essential for the following reasons:

(i) Long-term data are needed to investigate the long-term behaviour of different types of pumps because their lifetime depends on the pressure conditions they are exposed to over the years. These data are available through a web application for data plotting that uses the generated data from a database.

(ii) Short-term data (minutes to several days) are needed to investigate immediate problems in the vacuum systems (*e.g.* leakages, failures of pumps) and quick decisions on which measures need to be taken to solve problems. These data are available directly through the *Karabo* GUI.

A recent option for monitoring the system is the *Karabo* notification service: critical states of devices can be set as a threshold for sending an alarm and/or warning message to the vacuum responsible staff. Right now, this would be the vacuum beam permissions signal of the individual beamlines and the OK-state signal of all turbo pumps in the differential pumping sections.

## 7. Operational experience   

The experience from the commissioning phase and the first three years of operation confirms that the requirements regarding performance and reliability of the vacuum system, which were set during the design phase, were met. Only in a very few cases interruptions of beam delivery were caused by a problem related to the vacuum system; better statistics are required to give an accurate statement about the weak points of the system. Experience from regular maintenance that involved venting of a vacuum section, like exchange of a damaged solid attenuator foil, showed that recovering the vacuum can be achieved in less than 12 h. Practically, after pumping down the beamline section overnight with a pump cart, photon beam operation is usually possible the next day after a successful helium-leak test and switching back on the sputter ion pumps.

A combination of a preventive and predictive maintenance programme has been deployed for all the mechanical pumping systems that promote the high availability of this equipment and minimize the downtime due to functional failures. Also, a thoroughly estimated stock of spare components and servicing tools is available in order to enhance the intervention readiness capabilities.

The members of the vacuum group are continuously trained to further improve the availability of the vacuum system. To enable a quick reaction in case of failure, an on-call service has been set up that is available 24/7 and is staffed by experts from the vacuum group.

## 8. Conclusion and outlook   

**Figure 7:**
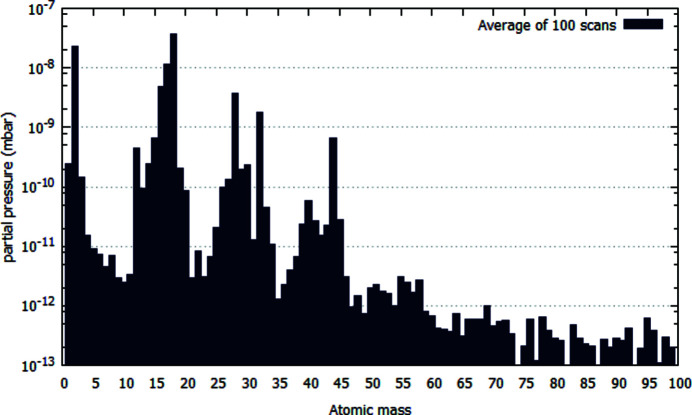
RGA spectra of a SASE3 distribution mirror chamber one day after installation shows that the amount of hydrocarbons present in the residual gas is below the required range.

In conclusion, the photon vacuum system of the European XFEL was shown to fulfil all parameters stated in the Technical Design Report (Sinn *et al.*, 2012 ▸). No particle contamination of X-ray mirror surfaces has been visible up to now. The establishment of particle-free areas of 30 m around the mirrors and gratings has been proven successful. Using mobile cleanrooms and particle counters to constantly monitor the environment during assembly has turned out to be indispensable in the particle free areas. Furthermore, the level of hydrocarbons in the residual gas has been checked by carrying out RGA measurements (Fig. 7 ▸) at various locations. The concentration is in a sensibly low range, which also confirms the appropriateness of the cleaning concept. First visual inspection of the mirrors of the soft X-ray beamline SASE3 shows only very light carbon deposition on the surfaces after more than two years of operation.

The gas attenuator meets all its design requirements and allows fast changes of attenuation levels.

PLC control software, user interface, interlock scheme and data archive allow easy and safe operation of the vacuum system and will be further developed in the upcoming years.

In a first planned upgrade of the whole system, we are aiming to increase the redundancy of the pressure reading by adding Pirani and cold cathode gauges to each section. Furthermore, additional NEG cartridge pumps will be installed to be better protected against power outages and failures of sputter ion pump high-voltage controllers. Ideally, each vacuum section shall host a NEG cartridge pump. A further ongoing project is the installation and commissioning of beam transport for the third scientific instrument at the SASE3 beamline, called the Soft X-ray Port (SXP), which will be completed and ready for commissioning with X-rays in 2022.

## Figures and Tables

**Table 1:** Summary of the most relevant characteristics of the SASE3 gas attenuator

Operation modes	‘Active absorption’ and ‘UHV’
Minimum transmission factor	<0.1% (for photon energies between 250 and 3200 eV)
Transmission factor range	Continuous from 1 × 10^−15^% up to 100%
Maximum operative pressure	Up to 35 mbar, regular operation up to 12 mbar
Minimum operative pressure	Down to 2 × 10^−3^ mbar
*P*_ultimate_ (UHV mode)	<6 × 10^−9^ mbar
Active gas cell dimensions	15 m (nominal) length × 100 mm diameter
Clear aperture variable system	Static: 20 mm; dynamic (selectable): 12 mm and 6 mm B_4_C apertures
Default gas species (purity)	Nitrogen (5.0) and argon (4.5)

**Table 2:** Overview of installed PLC hardware

	SASE1	SASE2	SASE3	SUM
Digital input (DI)	499	514	624	1637
Digital output (DO)	218	191	310	719
Analogue input (AI)	144	164	192	500
Serial interface	48	51	53	152
EtherCAT bus-coupler	36	46	42	124
